# *Mycolicibacterium smegmatis*, Basonym *Mycobacterium smegmatis*, Expresses Morphological Phenotypes Much More Similar to *Escherichia coli* Than *Mycobacterium tuberculosis* in Quantitative Structome Analysis and CryoTEM Examination

**DOI:** 10.3389/fmicb.2018.01992

**Published:** 2018-09-11

**Authors:** Hiroyuki Yamada, Masashi Yamaguchi, Yuriko Igarashi, Kinuyo Chikamatsu, Akio Aono, Yoshiro Murase, Yuta Morishige, Akiko Takaki, Hiroji Chibana, Satoshi Mitarai

**Affiliations:** ^1^Department of Mycobacterium Reference and Research, The Research Institute of Tuberculosis, Japan Anti-Tuberculosis Association, Kiyose, Japan; ^2^Medical Mycology Research Center, Chiba University, Chiba, Japan; ^3^Department of Basic Mycobacteriology, Graduate School of Biomedical Sciences, Nagasaki University, Nagasaki, Japan

**Keywords:** *Mycolicibacterium smegmatis*, *Mycobacterium tuberculosis*, structome analysis, cryo-fixation, freeze-substitution, serial ultrathin sectioning, transmission electron microscopy, ribosome enumeration

## Abstract

A series of structome analyses, that is, quantitative and three-dimensional structural analysis of a whole cell at the electron microscopic level, have already been achieved individually in *Exophiala dermatitidis, Saccharomyces cerevisiae, Mycobacterium tuberculosis*, Myojin spiral bacteria, and *Escherichia coli*. In these analyses, sample cells were processed through cryo-fixation and rapid freeze-substitution, resulting in the exquisite preservation of ultrastructures on the serial ultrathin sections examined by transmission electron microscopy. In this paper, structome analysis of non pathogenic *Mycolicibacterium smegmatis*, basonym *Mycobacterium smegmatis*, was performed. As *M. smegmatis* has often been used in molecular biological experiments and experimental tuberculosis as a substitute of highly pathogenic *M. tuberculosis*, it has been a task to compare two species in the same genus, *Mycobacterium*, by structome analysis. Seven *M. smegmatis* cells cut into serial ultrathin sections, and, totally, 220 serial ultrathin sections were examined by transmission electron microscopy. Cell profiles were measured, including cell length, diameter of cell and cytoplasm, surface area of outer membrane and plasma membrane, volume of whole cell, periplasm, and cytoplasm, and total ribosome number and density per 0.1 fl cytoplasm. These data are based on direct measurement and enumeration of exquisitely preserved single cell structures in the transmission electron microscopy images, and are not based on the calculation or assumptions from biochemical or molecular biological indirect data. All measurements in *M. smegmatis*, except cell length, are significantly higher than those of *M. tuberculosis*. In addition, these data may explain the more rapid growth of *M. smegmatis* than *M. tuberculosis* and contribute to the understanding of their structural properties, which are substantially different from *M. tuberculosis*, relating to the expression of antigenicity, acid-fastness, and the mechanism of drug resistance in relation to the ratio of the targets to the corresponding drugs. In addition, data obtained from cryo-transmission electron microscopy examination were used to support the validity of structome analysis. Finally, our data strongly support the most recent establishment of the novel genus *Mycolicibacterium*, into which basonym *Mycobacterium smegmatis* has been classified.

## Introduction

*Mycolicibacterium smegmatis* is a rapid-growing bacterium and previously belonged to the genus *Mycobacterium* as basonym *Mycobacterium smegmatis*, to which many pathogenic mycobacteria, including *M. tuberculosis*, a causative agent of tuberculosis, and *M. leprae*, a causative agent of leprosy, are belonging (Gupta et al., 2018; Oren and Garrity, 2018). Therefore, *M. smegmatis* has been quite often used as substitute for *M. tuberculosis* or *M. leprae* in studies, especially in the field of molecular biology, and there are a number of literature (Bashiri and Baker, 2015; Brown-Elliott and Philley, 2017), more than 15 papers, listed only in 2018 (Angara et al., 2018; Burian and Thompson, 2018; Chandran et al., 2018; Chen et al., 2018; Dal Molin et al., 2018; Ghosh et al., 2018; Goins et al., 2018; Jesus et al., 2018; Kaur et al., 2018; Kumar et al., 2018; Lopez et al., 2018; Marney et al., 2018; Mortuza et al., 2018; Richards et al., 2018; Singh et al., 2018; Tsaloglou et al., 2018; Verma et al., 2018).

We have already reported structome analysis data on *Exophiala dermatitidis* (Yamaguchi, 2006), *Saccharomyces cerevisiae* (Yamaguchi et al., 2011), *M. tuberculosis* (Yamada et al., 2015), Myojin spiral bacteria (Yamaguchi et al., 2016b), *Escherichia coli* (Yamada et al., 2017), and Myojin amorphous bacteria (Yamaguchi et al., 2018). In these previous studies, samples were prepared through rapid-freezing and freeze-substitution, and fundamental quantitative data of the single cells were provided with examination of serial ultrathin sections by transmission electron microscope (TEM), including cell diameter, length, volume of whole cell and cytoplasm, surface area, and cytoplasmic ribosome number. Because yeast cells have a larger cell volume, they express a higher number of total cytoplasmic ribosomes. However, *E. coli* has much higher ribosome density despite lower total ribosome number contained in a much smaller cytoplasm. In contrast, *M. tuberculosis*, Myojin spiral bacteria, and Myojin amorphous bacteria have much lower cytoplasmic ribosome density with lower total ribosome number in a much smaller cytoplasmic volume.

In this study, *M. smegmatis* structome analysis was done on seven cells, which were contained in serial ultrathin sections that spanned from one end to the other of the cell. The analysis was performed in the same manner so to compare it with *M. tuberculosis* structome data due to the fact that *M. smegmatis* of lower pathogenicity has been used as a substitute for the highly pathogenic *M. tuberculosis* in a large number of molecular biological or molecular genetic experiments. Further to this analysis, it was revealed that *M. smegmatis* had a significantly larger cell volume, both whole cell and cytoplasm, and significantly higher ribosome number and ribosome density than *M. tuberculosis*. *M. smegmatis* is seen to be similar to *E. coli*, rather than to *M. tuberculosis*, for these parameters. Upon comparing with *M. tuberculosis* cells, no significant difference was found in cell length alone.

In addition, as shown in the following text, using exquisite TEM images obtained from serial ultrathin sections, three-dimensional reconstructions were performed. In the reconstruction process, the ribosome distribution in each of the cytoplasm was clearly depicted in addition to the cell profiles. This is the first report on the three-dimensional reconstruction and ribosome-density enumeration of *M. smegmatis* cells based on TEM examination of serial ultrathin sections as well as ice-embedded whole mount cryoTEM observations.

## Materials and methods

### Bacteria

*M. smegmatis* (ATCC 19420) and *M. tuberculosis* H37Rv strain (ATCC 27294) were cultured in 50 ml of Middlebrook 7H9 (Becton Dickinson, Sparks, MD, USA), supplemented with oleic acid, bovine albumin (Fraction V), dextrose, and catalase (OADC, Becton Dickinson) enrichment and 0.05% Tween 80 (Sigma-Aldrich) contained in a 125-ml Erlenmeyer flask with a plain bottom (Nalgene, 4112-0125, NY, USA). Cells in the exponential growth phase were used. Aliquots (1 ml) of cultured cells were transferred directly to sterile microcentrifuge tubes without washing with buffer solution and centrifuged at 10,000 × *g* for 1 min. Normally, 6 ml of cultured cell suspension was used. The supernatants were discarded, and the remaining pellets were collected in two microcentrifuge tubes.

### Cryo-fixation, rapid freeze-substitution, and epoxy resin embedding

The sandwich method was performed as described previously (Yamaguchi, 2006; Yamada et al., 2010, 2015, 2017; Yamaguchi et al., 2011). Briefly, a portion (< 1 μl) of the highly concentrated bacterial pellet, prepared as described above was applied to a glow discharge-treated single-hole copper grid (Veco; hole size, 0.1-mm diameter) (Yamaguchi et al., 2016a) and then sandwiched with another glow discharge-treated single-hole grid. The grids were then picked up with tweezers and frozen by plunging them into mixture of melting propane and ethane cooled with liquid nitrogen with Vitrobot Mark IV (FEI, Thermo Fischer Scientific, USA). The pair of grids was transferred, detached in liquid nitrogen, and immersed quickly in 2% osmium tetroxide/acetone solution and then placed in the device described earlier and cooled. Next, the samples were transferred from the bio-safety facility and placed in a freezer at −85°C for several days, after which they were allowed to come to room temperature over several days in a conventional area of the laboratory. Then, the osmium tetroxide/acetone solution, was discarded, and the samples were washed with absolute acetone three times at room temperature. The samples were then embedded in Spurr's resin using Embedding Capsule Easy Molds (8-mm diameter, Leica/LKB) and polymerized at 70°C for 16 h.

### Preparation of serial ultrathin sections and TEM examination

Nearly 550 serial ultrathin sections with an average thickness of 40 nm were cut with an Ultracut E ultramicrotome (Reichert-Jung Co., Vienna, Austria) equipped with a diamond knife and then picked up using 10 single-slot copper grids with a slot size of 2.0 × 1.0 mm (Maxtaform HF49, Tonbridge, UK). The serial ultrathin sections were then transferred onto a formvar support film mounted on an aluminum rack with 20 pores of 4-mm diameter. Then, the grids with the sections were then dried, detached from the rack with the support of the formvar, and stained with uranyl acetate and lead citrate (Yamaguchi et al., 2009; Yamaguchi and Chibana, 2018). Of the 10 grids prepared, serial 3 grids were subjected to TEM examination. Among these 3 grids, serial ultrathin sections containing 7 cells on one grid were examined by TEM.

The TEM examinations were performed using a JEOL JEM-1230 electron microscope operated at 120 kV equipped with OSIS MegaView G2 (1K × 1K) CCD digital camera system (Olympus, Japan). Serial sections spanning the cell from one end to the other were searched at low magnification. Next, 7 cells in a total of 233 serial ultrathin sections were examined at higher magnification ( × 30,000, × 60,000, or × 80,000). Images were captured using iTEM software (OLYMPUS Soft Imaging Solutions GmbH, Münster, Germany).

### Whole-mount ice-embedded cryoTEM examination

Part of *M. smegmatis* and *M. tuberculosis* prepared as described earlier were fixed with 2.5% glutaraldehyde in phosphate buffer (0.1 M, pH 7.4) overnight at 4°C, and rinsed 3 times with distilled water. Two μL of the suspension was applied to a glow-discharged carbon grid with holes (Quantifoil® copper grids R2/1, Quantifoil MicroTools, Jena, Germany) and mounted in an environmentally controlled chamber at 100% humidity. The excess water was removed by blotting, and the grids were frozen in vitreous ice by plunging them into liquid ethane cooled with liquid nitrogen using a Vitrobot Mark II (FEI, Hillsboro, OR, USA). The grid was then transferred to a JEM-2200FS (JEOL) cryo-TEM equipped with a field emission gun. The microscope was operated at 200 kV acceleration voltage, and samples were cooled during examination with liquid nitrogen in the single-tilt nitrogen cryotransfer holder (Model 626, Gatan Inc., Pleasanton, CA. USA) at−175.9 to −177.8°C. Data were recorded with a 4k × 4k on-axis CCD camera (F415MP, TVIPS, Gauting, Germany), operated in a 2 × 2 hardware binning mode producing 2k × 2k images. Focusing and tracking were performed with a 1k × 1k off-axis CCD camera (Model F114, TVIPS) working in a continuous 1-frame/s animated acquisition mode, combined with a real-time Fourier transform display. Sample preparation after glutaraldehyde fixation and examination with cryoTEM were performed in the National Institute of Physiological Science (Okazaki, Aichi, Japan).

### Image analysis and ribosome enumeration

Images were saved as TIFF files and analyzed using ImageJ and Fiji software (Schindelin et al., 2012). Briefly, cell length was calculated by multiplying the number by 55 nm (representing the thickness of each section). The diameter (minor and major axes), perimeter, and thickness of the plasma membrane (PM), outer membrane (OM), and cell envelope of each cell were measured as a pixel value, using the line selection menu in the ImageJ/Fiji window as well as a scale bar recorded on the same negative. Measured pixel values were converted to μm or nm according to the measured pixel value of the scale bar on the corresponding negatives.

The cross-sectional area of each cell was determined using the “Measure” command in the “Analyze” menu of ImageJ/Fiji, by tracing the OM using the polygonal selection menu in the ImageJ window and converting the area result mentioned into μm^2^ by multiplying the square of the ratio of scale (nm) on the scanned negative by its pixel value. The cross-sectional area of each cell's cytoplasm was determined by tracing the PM in a like manner. The OM and PM surface areas (μm^2^) were calculated as the cumulative area of a trapezium of the cell in each section using the formula for calculating the area of a trapezoid, where the perimeter of the OM and PM in a given section and the previous section were used as the upper base and lower base, respectively, and the section thickness (0.040 μm [40 nm]) was used as the height.

The volume (fl, = μm^3^) of each cell was calculated as the cumulative volume of three-dimensional bodies having the OM- or PM-lined cross-sectional area as the base and the section thickness [0.040 μm [40 nm]] as the height for the whole cell volume and cytoplasmic volume, respectively. The volume of the periplasm was calculated by subtracting the cytoplasmic volume from the whole cell volume.

Ribosomes in the cytoplasm of the cell cross-section were enumerated using the “Multi-point Tool” in ImageJ/ Fiji (Schindelin et al., 2012). The total number of ribosomes in each cell and the number of ribosomes per 0.1 fl of cytoplasm were calculated based on the volume of each cell determined as described earlier.

### 3D reconstruction

Three-dimensional reconstruction was performed using TrackEM2 program contained in Fiji/ImageJ software version 2.0.0-rc-43 /1.50e; Java 1.6.0_65[64-bit] (Fiji, RRID:SCR_002285, ImageJ, RRID:SCR_003070) for seven cells. In addition, 3D reconstruction of Cell 3 was tried with Amira 6.5.0 software (Konrad-Zuse-Zentrum für Informationstechnik Berlin (ZIB), Germany, and FEI SAS, a part of Thermo Fisher Scientific).

### Statistics

Compared mean *t*-test and one-way ANOVA were performed to compare differences in mean values. Statistical analyses were performed using StatPlus:mac, AnalystSoft Inc.—statistical analysis program for Mac OS®. Version v6 (StatPlus Mac, RRID:SCR_014635).

## Results

### One-dimensional cell properties

**Figure 1:**
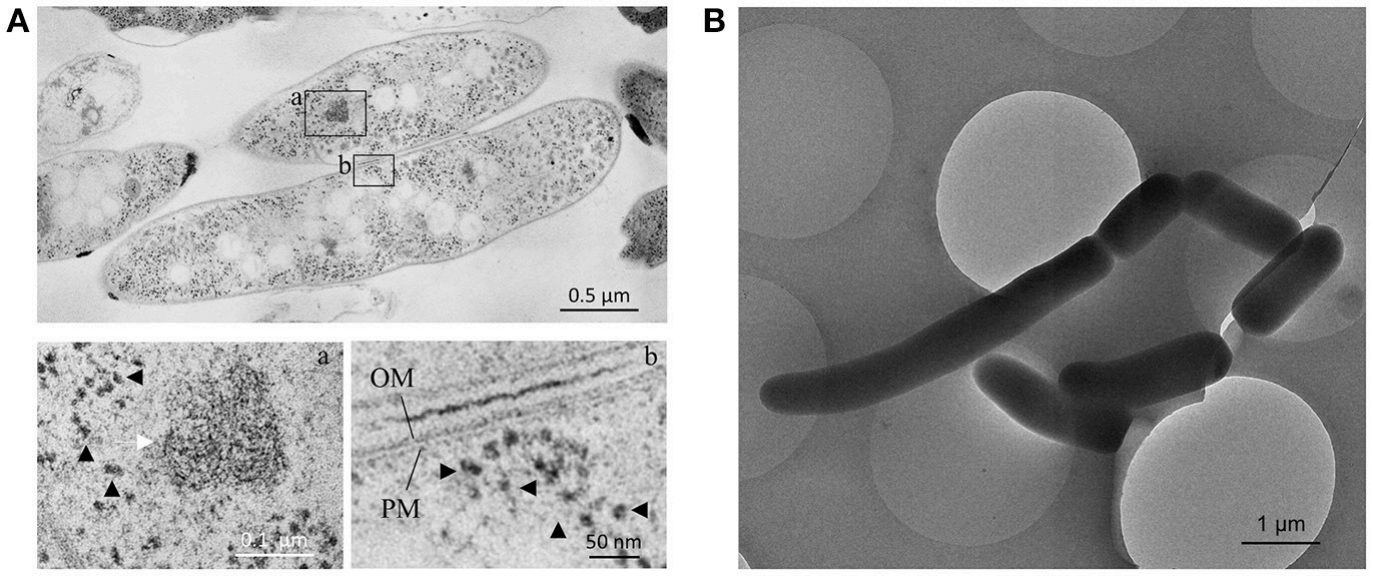
Images of ultrathin section and cryo-transmission electron microscopy (TEM) of *M. smegmatis* cells. **(A)** Two *M. smegmatis* cells processed through rapid-freeze and freeze-substitution in an ultrathin section. (a,b) enlarged images of the selected field of view in **(A)**. (a) Condensed DNA fiber (white arrow) and ribosome particles (black arrowhead). In (b) Ribosome particles (black arrowhead) and plasma membrane (PM) and outer membrane (OM). **(B)** CryoTEM image of *M. smegmatis* cells with varied cell lengths.

**Figure 2:**
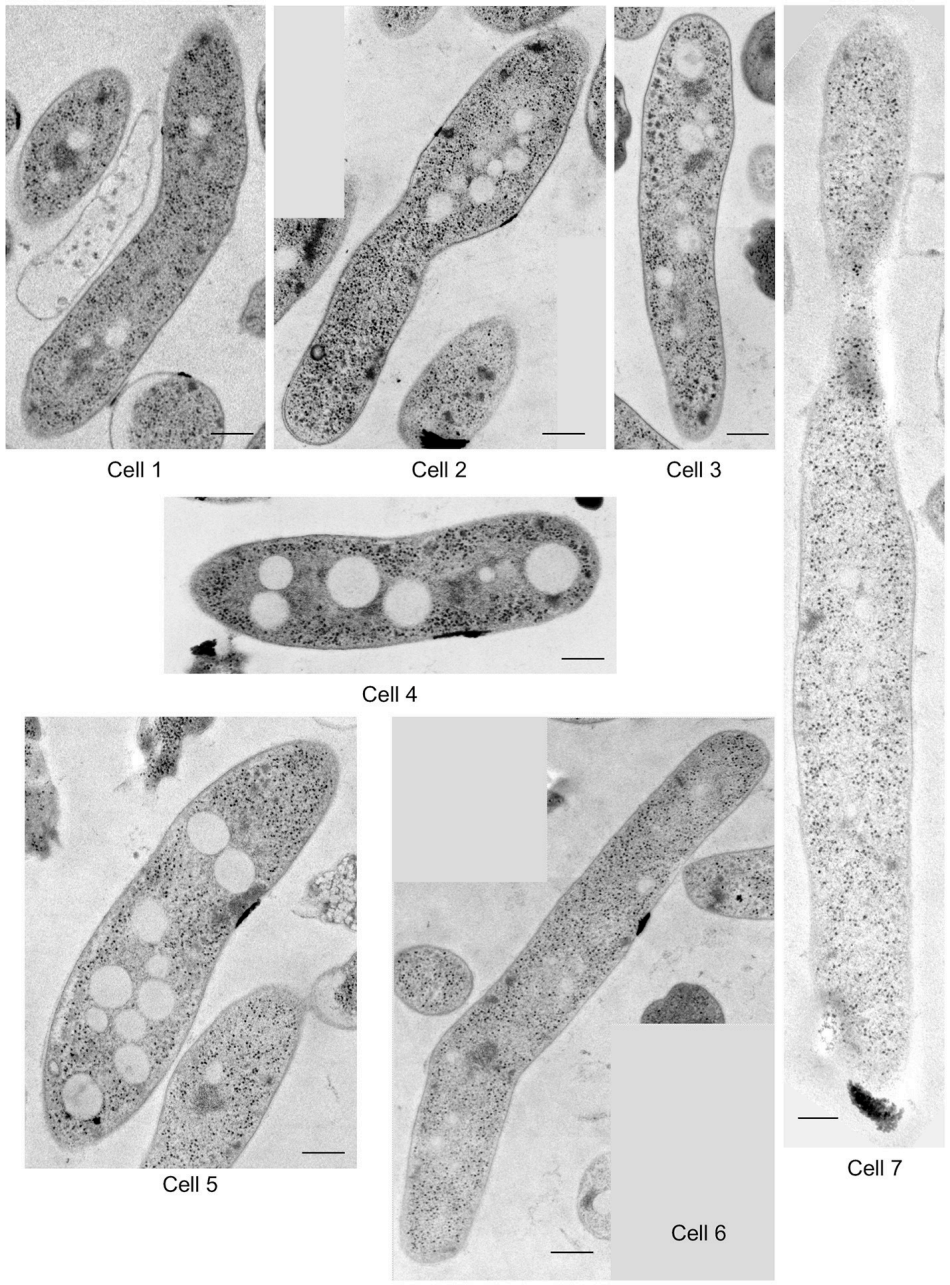
One of the representative images of serial ultrathin sections in seven *M. smegmatis* cells examined in structome analysis. Scale bar = 250 nm.

*M. smegmatis* cells were prepared through rapid-freezing and freeze-substitution to obtain much more exquisite cell images following epoxy resin embedding than those prepared through conventional chemical fixation (Figure 1A). With this preparation protocol, ultrastructures of cell were preserved with extremely high-quality, where not only membrane structures, but also every single ribosome can be examined and enumerated separately. In addition, ice-embedded whole-mount CryoTEM examination was performed (Figure 1B) to obtain cell profiles in the most intact condition. Seven *M. smegmatis* cells were examined on totally 220 serial ultrathin sections (Figure 2, Videos S1–S6, Table 1). Cell lengths of the seven cells ranged from 2.07 to 7.41 μm with average ± standard deviation (SD) as 3.54 ± 1.83 μm. On the contrary, cell length measured in CryoTEM examination for 61 *M. smegmatis* cells ranged from 1.08 to 6.27 μm, with average 3.46 ± 1.40 μm, which was similar to serial ultrathin section examination (Table S1).

**Table 1:** One-dimensional data of 7 *M. smegmatis* cells examined on serial ultrathin sections.

**Cell**	**Number of ultrathin sections examined**	**Length (μm)**	**Diameter (**μ**m)**		**Aspect ratio**
			**OM (Cell)**	**PM (Cytoplasm)**	
1	38	3.90	0.59	0.54	6.63
2	44	3.41	0.60	0.57	5.72
3	21	2.76	0.56	0.53	4.91
4	19	2.16	0.51	0.49	4.26
5	35	3.10	0.66	0.63	4.69
6	28	2.07	0.62	0.59	3.35
7	35	7.41	0.52	0.50	14.35
Average		3.54	0.58	0.55	6.27
SD		1.83	0.05	0.05	3.71
Min		2.07	0.51	0.49	3.35
Max		7.41	0.66	0.63	14.35

Cell diameter measured at OM and PM in serial ultrathin sections ranged from 0.51 to 0.66 and from 0.49 to 0.63, respectively, and the average values were 0.58 ± 0.05 μm and 0.55 ± 0.05 μm, respectively. In cell diameter, seven cells showed similar values to each other. Then, the standard deviations for both were small; less than 10% of averages (Table 1). On the contrary, cell diameter measured in CryoTEM examination ranged from 0.66 to 1.00 μm with an average of 0.77 ± 0.08 μm, which was significantly larger than that of serial ultrathin sections (0.58 ± 0.05 μm, *p* < 0.000001, Table S1). The aspect ratio of these seven cells was 6.27 ± 3.71.

These data were compared with those of *M. tuberculosis* and *E. coli* cells, including data obtained from CryoTEM examination (Table S1). In comparison of cell diameter, both average OM (0.58 ± 0.05 μm) and PM diameter (0.55 ± 0.05 μm) of *M. smegmatis* was significantly larger than that of *M. tuberculosis* (0.34 ± 0.03 μm for OM and 0.30 ± 0.02 μm for PM, *p* < 0.0001, respectively) and significantly smaller than that of *E. coli* (0.89 ± 0.06 μm, *p* < 0.0001). Furthermore, average whole cell diameter of *M. smegmatis* cell in cryoTEM (0.77 ± 0.08 μm) was significantly larger than that of *M. tuberculosis* (0.60 ± 0.06 μm, *p* < 0.0001). In cell length, whereas, there was no significant difference between the three species in the serial ultrathin sections, average length of *M. smegmatis* in cryoTEM (3.46 ± 1.40 μm) was significantly longer than that of *M. tuberculosis* (2.65 ± 0.74 μm, *p* < 0.05). In aspect ratio, average aspect ratio of *M. smegmatis* cells in serial ultrathin sections (6.27 ± 3.71) was significantly higher than that of *E. coli* (2.84 ± 0.46, *p* < 0.001), whereas there was no significant difference when compared with that of *M. tuberculosis* (8.24 ± 3.61, *p* > 0.3).

In addition, *M. smegmatis* expresses different morphological phenotypes from *M. tuberculosis* in Ziehl-Neelsen staining, and Gram staining, (Figure S1). In Ziehl-Neelsen staining, *M. smegmatis* cells easily lost acid-fastness, and most cells were positive in Gram staining, whereas most *M. tuberculosis* cells preserved acid-fastness vividly and varied in stainability from cell to cell in Gram staining (Figure S1). Difference in acid-fastness between *M. tuberculosis* and *M. smegmatis* is attributable to the differential expression of varied types of mycolic acid. That is, α-*cis/cis*, methoxy *cis*, methoxy *trans*, keto *cis*, and keto *trans* mycolic acids are primary contained in the cell wall of *M. tuberculosis*, whereas the cell wall of *M. smegmatis* primarily comprises of α-trans/trans, α'-*cis*, and epoxy *trans* mycolic acids. The α-trans/trans, α'-*cis*, and epoxy *trans* mycolic acids in *M. smegmatis* have less carbon and shorter chain lengths than those of mycolic acids contained in the cell wall of *M. tuberculosis* (Bhatt et al., 2005; Marrakchi et al., 2008; Vilvhéze and Kremer, 2017).

### Two-dimensional cell properties

Two-dimensional cell properties were measured. Using the perimeter value of OM and PM measured by Fiji/ImageJ, total areas of both surfaces were calculated. In seven *M. smegmatis* cells embedded in serial ultrathin sections, the average OM and PM surface areas were 5.62 ± 2.19 μm^2^ and 5.10 ± 1.95 μm^2^, respectively (Table 2). Both surface areas of *M. smegmatis* were significantly larger than that of *M. tuberculosis*, 3.03 ± 1.33 μm^2^ and 2.67 ± 1.19 μm^2^, respectively (both *p* < 0.05, Table S2). There were no significant differences in comparison of surface areas between *M. smegmatis* and *E. coli* or between *M. tuberculosis* and *E. coli* (Table S2).

**Table 2:** Two-dimensional data of 7 *M. smegmatis* cells examined on serial ultrathin sections.

**Cell**	**Surface area (**μ**m**^**2**^**)**	
	**OM**	**PM**
1	7.08	6.18
2	6.24	5.31
3	4.25	3.89
4	3.00	2.77
5	5.89	5.71
6	3.57	3.37
7	9.28	8.48
Average	5.62	5.10
SD	2.19	1.95

### Three-dimensional cell properties

Three-dimensional cell properties of seven *M. smegmatis* cells were measured and calculated as described in Materials and Methods (Table 3). Average whole cell volume was 0.91 ± 0.37 fl. This value was significantly larger than that of *M. tuberculosis* (0.29 ± 0.11 fl, *p* < 0.005, Table S3). Average cytoplasmic volume was 0.77 ± 0.31 fl. This value was also significantly larger than that of *M. tuberculosis* (0.21 ± 0.09 fl, *p* < 0.005, Table S3). The OM, PM, and periplasm volume in average were 0.01 ± 0.004 fl, 0.03 ± 0.01 fl, and 0.10 ± 0.07 fl, respectively, and there was no significant difference between *M. smegmatis* and *M. tuberculosis* (Table 3 and Table S3). Finally, there were no significant differences in all cell volume categories between *M. smegmatis* and *E. coli*. It is suggested that *M. smegmatis* cells are much more similar to *E. coli* cells than the cells of *M. tuberculosis*, which had belonged to the same genus (Table 3 and Table S3).

**Table 3:** Three-dimensional data of 7 *M. smegmatis* cells examined on serial ultrathin sections.

**Cell**	**Volume (fl)**				
	**Whole cell**	**OM**	**Periplasm**	**PM**	**Cytoplasm**
1	1.06	0.01	0.19	0.03	0.82
2	0.95	0.01	0.10	0.03	0.81
3	0.69	0.01	0.08	0.02	0.58
4	0.44	0.01	0.03	0.01	0.38
5	1.07	0.01	0.07	0.03	0.96
6	0.62	0.01	0.04	0.02	0.55
7	1.55	0.02	0.19	0.04	1.30
Average	0.91	0.01	0.10	0.03	0.77
SD	0.37	0.00	0.07	0.01	0.31

In addition, *M. smegmatis* express different morphological phenotypes from *M. tuberculosis* in cord formation as observed on the colonies grown on solid medium (Figure S2). The *M. tuberculosis* cells on solid medium expressed highly organized cord formation entirely, although the *M. smegmatis* colony expressed cord formation partially (Figure S2).

**Figure 3:**
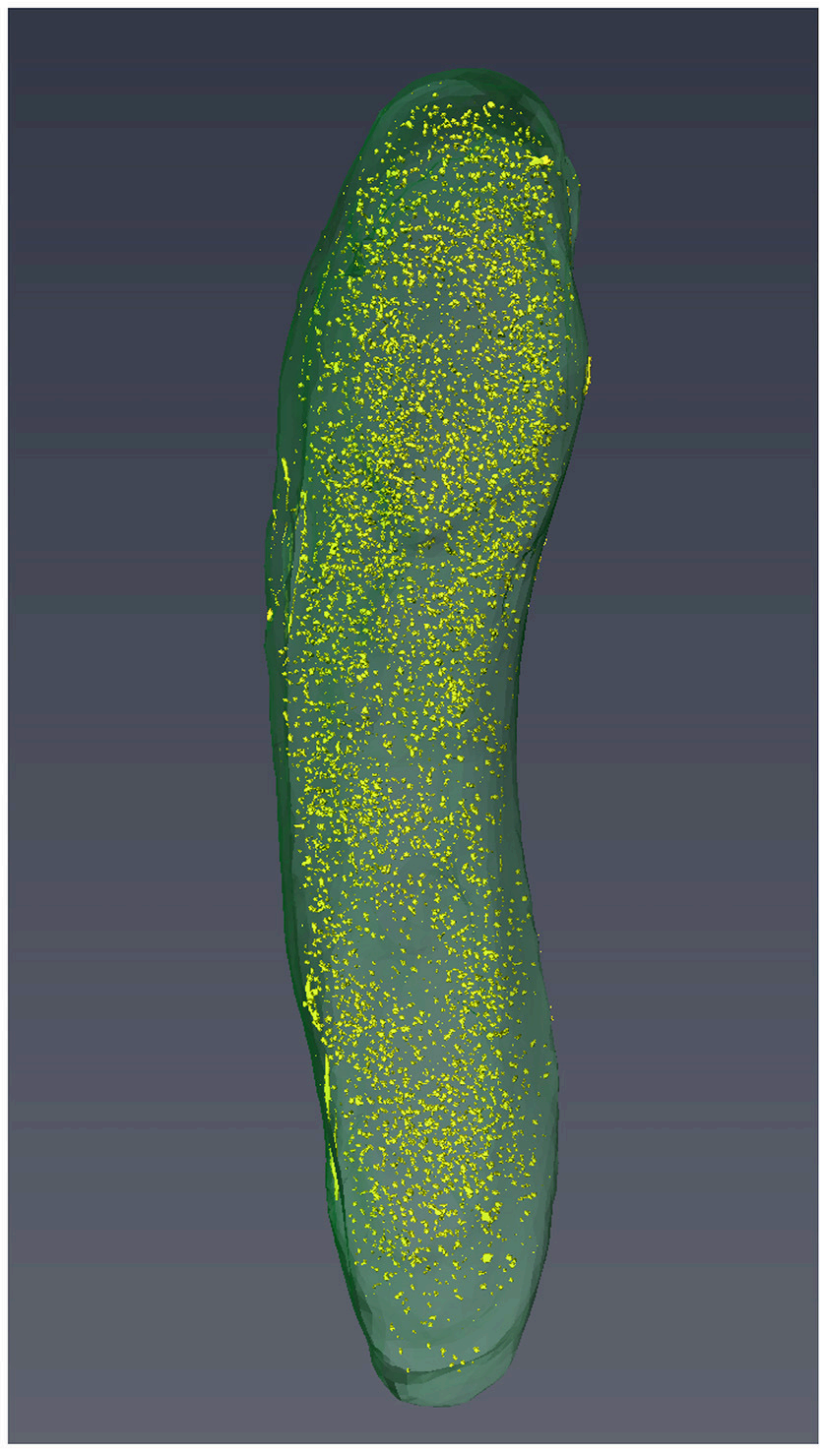
The 3D reconstruction of Cell 3 with visualization of cell profile and cytoplasmic distribution of ribosomes.

Three-dimensional reconstitutions for seven *M. smegmatis* cells were performed and these support the results obtained from structome analysis that cell length varied from cell to cell (Figure 3, Videos S7–S12).

### Ribosome enumeration

Cytoplasmic ribosome number was enumerated on each serial ultrathin section for seven cells. Total ribosome number per cell ranged from 5,430 to 12,930 with an average of 8,670 ± 2,660 (Table 4). On the contrary, ribosome density per 0.1 fl cytoplasm ranged from 990 to 1,420 with an average of 1,170 ± 180 (Table 4). As shown in Figure 3, ribosomes were evenly distributed throughout the cytoplasmic space in 3D reconstruction. The average total ribosome number of *M. smegmatis* (8,670 ± 2,660) was significantly larger than those of Myojin spiral bacteria (320 ± 120, *p* < 0.00001) and *M. tuberculosis* (1,670 ± 570, *p* < 0.0002), but significantly smaller than *E. coli* (26,210 ± 4,020, *p* < 0.000001), *E. dermatitidis* (195,000 ± 91,100, *p* < 0.0005), and *S. cereviciae* (195,000 ± 54,800, *p* < 0.000002, Table 5). In addition, average ribosome density of *M. smegmatis* (1,170 ± 180/0.1 fl cytoplasm) was significantly higher than Myojin spiral bacteria (220 ± 120/0.1 fl cytoplasm, *p* < 0.000005), Myojin amorphous bacteria (310 ± 40/0.1 fl cytoplasm, *p* < 0.000005), and *M. tuberculosis* (720 ± 170/0.1 fl cytoplasm, *p* < 0.005), but significantly lower than *E. coli* (2,840 ± 120/0.1 fl cytoplasm, *p* < 0.000001) and *S. cerevisiae* (1,950 ± 100/0.1fl cytoplasm, *p* < 0.000001, Table 5).

**Table 4:** Total ribosome number and cytoplasmic ribosome density of 7 *M. smegmatis* cells examined on serial ultrathin sections.

**Cell**	**Ribosome enumeration**		
	**Total number per cell**	**Density per 0.1 fl cytoplasm**	**Number per section**
1	9,290	1,140	245
2	8,580	1,060	150
3	8,210	1,420	390
4	5,430	1,420	290
5	10,630	1,110	300
6	5,620	1,020	200
7	12,930	990	370
Average	8,670	1,170	280
SD	2,660	180	90

**Table 5:** Comparison of total ribosome number and cytoplasmic density in structome-analyzed microorganisms.

**Microorganism**	**Number of cells examined**	**Ribosome enumeration**		
		**Average total ribosome number**	**Cytoplasmic volume (fl)**	**Average density (per 0.1 fl cytoplasm)**
Myojin spiral bacteria^1^	6	320 ± 120^a^	0.18 ± 0.07	220 ± 120^f^
Myojin amorphous bacteria^2^	10	1, 150 ± 370^b^	0.37 ± 0.09	310 ± 40^f^
*M. tuberculosis*^3^	5	1, 670 ± 570^b^	0.21 ± 0.09	720 ± 170^g^
*M. smegmatis*	7	8, 670 ± 2, 660	0.77 ± 0.31	1, 170 ± 180
*E. coli*^6^	9	26, 120 ± 4, 000^c^	0.90 ± 0.16	2, 840 ± 120^h^
*E. dermatitidis*^4^	5	195, 000 ± 91, 100^d^	17.1 ± 6.3	1, 100 ± 150
*S. cerevisiae*^5^	6	195, 000 ± 54, 800^e^	10.0 ± 2.6	1, 950 ± 100^h^

a *p < 0.00001*.

b *p < 0.0002*.

c *p < 0.000001*.

d *p < 0.0005*.

e *p < 0.000002*.

f *p < 0.000005*.

g *p < 0.005*.

h *p < 0.000001*.

1 *Yamaguchi et al. (2016b)*.

2 *Yamaguchi et al. (2018)*.

3 *Yamada et al. (2015)*.

4 *Yamaguchi (2006)*.

5 *Yamaguchi et al. (2011)*.

6 *Yamada et al. (2017)*.

## Discussion

### One-dimensional cell property

As shown in Table S1, the cell diameters of *M. smegmatis* cells examined with both serial ultrathin sections and cryoTEM were significantly larger than those of *M. tuberculosis* cells. In addition, cell lengths of *M. smegmatis* cells examined with cryoTEM were significantly longer than those of *M. tuberculosis* cells. In comparison with *E. coli* cells examined with serial ultrathin sections, the average diameter of *M. smegmatis* cells was significantly smaller than those of *E. coli*, whereas there was no significant difference in average cell length between *M. smegmatis* and *E. coli*.

As discussed in previous literature, cell division of *M. tuberculosis* cells may occur without recognition of the middle of the cell long axis because the length of the longest cell was much more than twice of the length of the shortest cell (Yamada et al., 2015). This phenomenon is much more obvious in *M. smegmatis* cells because the length of the longest cell was three times and six times longer than the shortest cell in serial ultrathin sections and CryoTEM examinations, respectively (Table S1). Asymmetric cell divisions in *M. smegmatis* cell were reported previously. In a single cell time-lapse observation, Aldridge *et al*. examined the difference in elongation of two poles of the growing single cell, where old, that is, mother's pole elongates faster than new or young pole, which has been generated during the previous division. Then, cells with older growth poles elongate faster than cells with younger growth poles. In addition, the birth length of cells increases as the growth pole matures. Taken together, these data suggest that as the growth pole matures, cells elongate faster and are larger (Aldridge et al., 2012, p.1).

Furthermore, Vijay *et al*. also examined the time-lapse differential interference contrast microscopy of *M. smegmatis* cells, and demonstrated that about 20% of the septating *M. smegmatis* cells in the exponential phase population divided asymmetrically and generated two daughter cells having different cell lengths (Vijay et al., 2014). The authors confirmed the phenomena by TEM examination. Our data confirm these reports. On the contrary, the length of *E. coli* cells in serial ultrathin sections ranged from 2.05 to 3.04, where the shortest cell length was two-third (Yamada et al., 2017). This result indicates that *E. coli* divide into equally half, and the examined cell in serial ultrathin sections did not contain cells just divided (Table S1). It revealed that the machinery of cell division in mycobacteria completely differs from that of *E. coli*.

### Two-dimensional cell properties

This is the first report on the surface area of the *M. smegmatis* cells. The OM and PM cell surface areas of seven *M. smegmatis* cells ranged from 3.00 to 9.28 μm^2^, with an average of 5.62 ± 2.19 μm^2^ and ranged from 2.77 to 8.48 μm^2^, with an average of 5.10 ± 1.95 μm^2^, respectively. These values are approximately twice of those of *M. tuberculosis*, and the differences were significant (*p* < 0.05, Table S2). This suggests that if *M. tuberculosis* cell surface antigens expressed in *M. smegmatis* cells, the numbers of antigen molecules in single *M. smegmatis* cell may double at least compared to those of *M. tuberculosis*. In the circumstance, the gene(s) from *M. tuberculosis* transferred *M. smegmatis* cannot be used as a real substitute of *M. tuberculosis* because of the difference in substantial number of antigen molecules in a single cell even if the two species belong to the same genus and share a phyletic affinity. There were no significant differences between *M. smegmatis* and *E. coli*, suggesting that *M. smegmatis* cells may be much more similar to *E. coli* cells morphologically than *M. tuberculosis* cells.

### Three-dimensional cell properties

Average whole cell and cytoplasmic volume of *M. smegmatis* cells in serial ultrathin sections were 0.91 ± 0.37 fl and 0.77 ± 0.31 fl, respectively (Table 3). These values are approximately three times larger than those of *M. tuberculosis*, and the differences were significant (*p* < 0.005, Table S3). It is suggested that *M. tuberculosis*-gene transferred *M. smegmatis* cell can contain three times more molecules than the parental *M. tuberculosis* cell. Based on these results, if *in vivo* or *in vitro* experiments are performed using *M. tuberculosis*-gene transferred *M. smegmatis*, interpretation of the results should be considered carefully and should not be directly extrapolated to *M. tuberculosis* infection. In the cell volume analysis in serial ultrathin sections, there were no significant differences between *M. smegmatis* and *E. coli* (Table S3).

Together with surface areas of OM and PM described earlier, the volume of OM, periplasm, and PM of *M. smegmatis* cells were calculated from perimeter and serial ultrathin section thickness in TEM images. Compared with the *M. tuberculosis*, the volume of the perimeter and PM of *M. smegmatis* cells were 1.5 times larger than *M. tuberculosis*, but the differences were not significant. These evidences suggest that single *M. smegmatis* cells can contain a maximal of 1.5 times more molecules in the periplasm and in the PM than *M. tuberculosis* cells.

### Total enumeration and cytoplasmic density of ribosomes

Structome analysis data have been already reported in six species, including two yeast species and four bacterial species (Table 5) (Yamaguchi, 2006; Yamaguchi et al., 2011, 2016b, 2018; Yamada et al., 2015, 2017). Because total ribosome number per cell can differ from cell to cell according to the volume of cytoplasm with a large standard deviation, even if the cells would belong to the same strain, the value is not appropriate to be used in comparison between species or strains.

**Figure 4:**
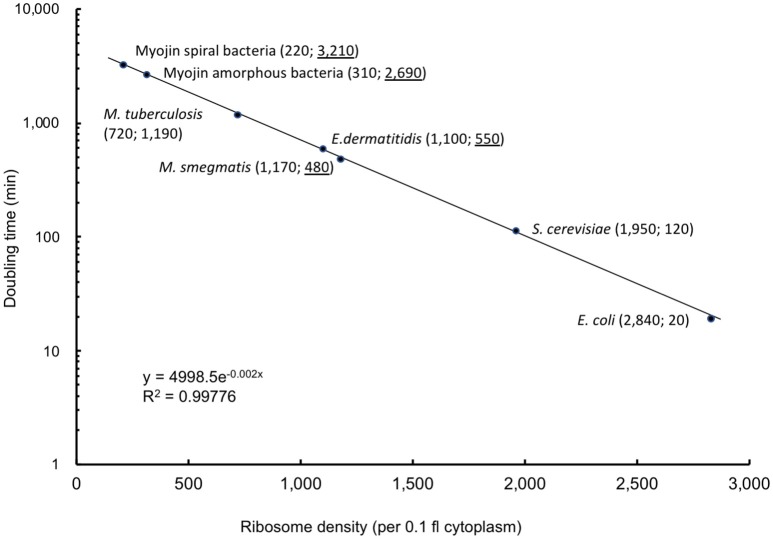
Correlation curve between ribosome density per 0.1 fl cytoplasm and doubling time (min). Correlation curve was drawn based on known doubling time and ribosome densities per 0.1 fl cytoplasm enumerated in previous structome analysis of *E. coli, M. tuberculosis*, and *S. cereviciae*. Number in parenthesis indicates ribosome density per 0.1 fl cytoplasm (left) and doubling time (min) (right). Underlined doubling time (min) in species with unknown doubling time, Myojin spiral bacteria, Myojin amorphous bacteria, *E. dermatitidis*, and *M. smegmatis* were calculated based on the formula y = 4998.5e^−0.002x^.

On the contrary, it has been revealed that ribosome density per unit volume of cytoplasm is specific to the species or the strain with an extremely small standard deviation (Table 5) (Yamaguchi, 2006; Yamaguchi et al., 2011, 2016b, 2018; Yamada et al., 2015, 2017). Average total cytoplasmic ribosome number of *M. smegmatis* cells was 8,670 ± 2,660. This value was significantly more than Myojin spiral bacteria and *M. tuberculosis*, but significantly less than *E. dermatitidis, S. cerevisiae*, and *E. coli* (Table 5). However, average cytoplasmic ribosome density of *M. smegmatis* cells was 1,170 ± 180 per 0.1 fl cytoplasm, and this value was significantly more than Myojin spiral bacteria, Myojin amorphous bacteria, and *M. tuberculosis*, but significantly less than *S. cerevisiae* and *E. coli*. It is then surprising that there was only a small difference in average cytoplasmic ribosome density between *M. smegmatis* and yeast *E. dermatitidis*, which had nearly 200,000 total ribosomes in the cytoplasm (Yamaguchi, 2006, Table 5 and Figure 4). *M. smegmatis* cells had volume of cytoplasm and ribosome density much more similar to *E. coli* cells than *M. tuberculosis* cells. These results supported that *M. smegmatis* and *E. coli* have multiple copy number of *rrn*, gene encoding rRNA, whereas *M. tuberculosis* has only a single copy (Gonzalez-y-Merchand et al., 1999; Roller et al., 2016).

Srivastava et al. established an approach to reconstitute mycobacterial protein translation in *E. coli* cells *in vitro* (Srivastava et al., 2016). Authors used *M. tuberculosis* translation factors (TF), *M. smegmatis* aminoacyl tRNA synthetases (AARSs) mixture, *M. smegmatis* tRNAs, *M. smegmatis* ribosomes, *E. coli* energy regeneration enzymes, *E. coli* methionyl tRNA formyltransferase, T7 RNA polymerase, and some small molecules and buffers. When ribosomes of *M. smegmatis* and tRNA, AARSs, and TF of *E. coli* were used, the relative reporter activity was nearly 30% of complete *E. coli* cells. The evidence supports our data that the cytoplasmic ribosome density of *M. smegmatis* was one-third of that of *E. coli* (Table 5). However, Srivastava et al. used ribosomes at a molar concentration of 2.4 μM (Srivastava et al., 2016). This value means 1.4 × 10^11^ ribosomes molecules per 0.1 fl, which is 10^8^ and 10^7^ times more density than the actual ribosome density per 0.1 fl cytoplasm of *M. smegmatis* and *E. coli* cells, respectively. It is intriguing that this huge number of ribosomes may be acceptable in experimental conditions.

On the contrary, it has been demonstrated that *M. smegmatis* ribosomes have a unique structural property in 3D architecture through cryo-TEM mapping (Shasmal and Sengupta, 2012; Yang et al., 2017). Compared with *E. coli* ribosome proteins, 11 ribosome proteins of *M. smegmatis* were longer than those of *E. coli*, with more than 14 amino acid extensions.

As mentioned in our previous paper, there is a close correlation between ribosome density and doubling time (min) (Yamada et al., 2017). According to data obtained from this study, the correlation chart was revised as shown in Figure 4 based on the known doubling time data in *E. coli, S. cerevisiae*, and *M. tuberculosis* (Cox, 2004; Papagiannakis et al., 2017).

In *E. coli*, there are many studies that address the relationship between ribosome number and doubling times (Dennis, 1972; Dennis and Bremer, 1974; Cox, 2003; Bakshi et al., 2012; Milo and Phillips, 2016), which support the results of this study. On the contrary, in *M. smegmatis*, there are few studies that have examined the relationship between ribosome density and the doubling time. Liu et al. observed ribosome content, not ribosome density, in the ultrathin sections of chemically fixed samples, which were genetically engineered to overexpress Rv2629 (Liu et al., 2017). The data cannot be compared with our data because our samples were prepared through rapid-freezing and freeze-substitution, and ribosome density was calculated by direct enumeration of cytoplasmic ribosome number and volume of cytoplasm in serial ultrathin sectioning spanning a cell from one end to the other end.

Furthermore, Zhu and Dai reviewed the correlation of protein synthesis rate and ribosome content with growth rate in *E. coli*, mycobacterial species, and *S. cerevisiae* (Zhu and Dai, 2018). However, because these ribosome quantifications were described as ribosome content derived from a population of microorganisms, and not ribosome density obtained from direct enumeration through TEM examination of serial ultrathin sections in a single cell, quantifications were much less accurate than our structome analysis.

It is possible to estimate doubling time of viable, but non culturable microorganisms from cytoplasmic ribosome density obtained from structome analysis using the formula y = 4998.5e^−0.002x^, where x is ribosome density per 0.1 fl cytoplasm and y is doubling time (min), respectively.

## Conclusions

It is revealed that although *M. smegmatis* had belonged to the genus *Mycobacterium, M. smegmatis* is less similar to *M. tuberculosis* in the aspect of cell biology with significant differences, including acid-fastness, cell diameter, cell length, surface areas, cell volume, total ribosome number, and ribosome density. Most recently, genus *Mycobacterium* has been divided into an emended genus *Mycobacterium* and four novel genera, *Mycolicibacterium* gen. nov., *Mycolicibacter* gen. nov., *Mycolicibacillus* gen. nov., and *Mycobacteroides* gen. nov. corresponding to the “*Fortuitum-Vaccae,”* “*Terrae,”* “*Triviale*,” and “*Abscessus-Chelonae”* clades, respectively (Gupta et al., 2018; Oren and Garrity, 2018).

Non pathogenic *M. smegmatis* has been used as a surrogate of highly pathogenic *M. tuberculosis* because non pathogenic *M. smegmatis* can be easily and freely used in the biosafety level 1 laboratory, whereas highly pathogenic *M. tuberculosis* must be strictly manipulated in the biosafety level 3 laboratory. Single *M. smegmatis* cells expressing twice the number of *M. tuberculosis* antigens can elicit excess host cell responses compared with the native *M. tuberculosis*. The more the antigens that can be contained in their cell surface, the more the host cell response that can be elicited. In addition, *M. smegmatis* cell contains different mycolic acid species. This means that *M. smegmatis* cell can contain different antigenic molecules, both in quality and in quantity from *M. tuberculosis*. Therefore, it is not appropriate to use *M. smegmatis* as surrogate of *M. tuberculosis* in the investigation of the pathogenicity or virulence of *M. tuberculosis*. *M. smegmatis* has been classified to novel genus *Mycolicibacterium*, which is different from genus *Mycobacterium*. This means usage of *M. smegmatis* as a surrogate of *M. tuberculosis* is not appropriate. The *M. bovis* BCG strains are more appropriate to be used as surrogates despite of RD1 deletion, which must be manipulated in the biosafety level 2 facility.

We propose that data obtained from the experiments with *M. smegmatis* should be interpreted as those from a completely distinct species or genus from *M. tuberculosis*, and that it is critical to perform structome analysis aiming at the investigation of quantitative single-cell properties and the establishment of species-specific cell properties based on the ultrastructural images.

## Author contributions

HY and MY performed experiments. YI, KC, AA, YoM, YuM, AT, HC, and SM assisted in the experiments. HY and MY acquired data and HY and YI analyzed the data. HY performed the statistical analysis, and HY, MY, and SM wrote the manuscript.

### Conflict of interest statement

The authors declare that the research was conducted in the absence of any commercial or financial relationships that could be construed as a potential conflict of interest.

## Abbreviations

OM: outer membrane
PM: plasma membrane
TEM: transmission electron microscope.

## References

[B1] Aldridge B. B. Fernandez-Suarez M. Heller D. Ambravaneswaran V. Irimia D. Toner M.. (2012). Asymmetry and aging of mycobacterial cells lead to variable growth and antibiotic susceptibility. Science 335, 100–104. 10.1126/science.1216166 22174129 PMC3397429

[B2] Angara R. K. Yousuf S. Gupta S. K. Ranjan A. (2018). An IclR like protein from mycobacteria regulates leuCD operon and induces dormancy-like growth arrest in *Mycobacterium smegmatis*. Tuberculosis 108, 83–92. 10.1016/j.tube.2017.10.009 29523332

[B3] Bakshi S. Siryaporn A. Goulian M. Weisshaar J. C. (2012). Superresolution imaging of ribosomes and RNA polymerase in live *Escherichia coli* cells. Mol. Microbiol. 85, 21–38. 10.1111/j.1365-2958.2012.08081.x 22624875 PMC3383343

[B4] Bashiri G. Baker E. N. (2015). Production of recombinant proteins in *Mycobacterium smegmatis* for structural and functional studies. Protein Sci. 24, 1–10. 10.1002/pro.2584 25303009 PMC4489920

[B5] Bhatt A. Kremer L. Dai A. Z. Sacchettini J. C. Jacobs W. R. Jr. (2005). Conditional depletion of KasA, a key enzyme of mycolic acid biosynthesis, leads to mycobacterial cell lysis. J. Bacteriol. 187, 7596–7606. 10.1128/JB.187.22.7596-7606.2005 16267284 PMC1280301

[B6] Brown-Elliott B. Philley J. (2017). Rapidly growing mycobacteria. Microbiol. Spectr. 5:TNMI7-0027-2016. 10.1128/microbiolspec.TNMI7-0027-201 28084211 PMC11687460

[B7] Burian J. Thompson C. J. (2018). Regulatory genes coordinating antibiotic-induced changes in promoter activity and early transcriptional termination of the mycobacterial intrinsic resistance gene *whiB7*. Mol. Microbiol. 107, 402–415. 10.1111/mmi.13890 29205551

[B8] Chandran A. V. Jayanthi S. Vijayan M. (2018). Structure and interactions of RecA: plasticity revealed by molecular dynamics simulations. J. Biomol. Struct. Dyn. 36, 98–111. 10.1080/07391102.2016.1268975 28049371

[B9] Chen Y. Cao S. Sun Y. Li C. (2018). Gene expression profiling of the TRIM protein family reveals potential biomarkers for indicating tuberculosis status. Microb. Pathog. 114, 385–392. 10.1016/j.micpath.2017.12.008 29225091

[B10] Cox R. A. (2003). Correlation of the rate of protein synthesis and the third power of the RNA: protein ratio in *Escherichia coli* and *Mycobacterium tuberculosis*. Microbiology 149, 729–737. 10.1099/mic.0.25645-0 12634341

[B11] Cox R. A. (2004). Quantitative relationships for specific growth rates and macromolecular compositions of *Mycobacterium tuberculosis, Streptomyces coelicolor* A3(2) and *Escherichia coli* B/r: an integrative theoretical approach. Microbiology 150, 1413–1426. 10.1099/mic.0.26560-0 15133103

[B12] Dal Molin M. Gut M. Rominski A. Haldimann K. Becker K. Sander P. (2018). Molecular mechanisms of intrinsic streptomycin resistance in *Mycobacterium abscessus*. Antimicrob. Agents Chemother. 62, e01427–e01417. 10.1128/AAC.01427-17 29061744 PMC5740355

[B13] Dennis P. P. (1972). Regulation of ribosomal and transfer ribonucleic acid synthesis in *Escherichia coli* B/r. J. Biol. Chem. 247, 2842–2845. 4337104

[B14] Dennis P. P. Bremer H. (1974). Macromolecular Composition During Steady-State Growth of *Escherichia coli* B/r. J Bacteriol. 119, 270–281. 4600702 10.1128/jb.119.1.270-281.1974 PMC245599

[B15] Ghosh G. Reddy J. Sambhare S. Sen R. (2018). A bacteriophage capsid protein is an inhibitor of a conserved transcription terminator of various bacterial pathogens. J. Bacteriol. 200, e00380–e00317. 10.1128/JB.00380-17 29038252 PMC5717163

[B16] Goins C. M. Schreidah C. M. Dajnowicz S. Ronning D. R. (2018). Structural basis for lipid binding and mechanism of the *Mycobacterium tuberculosis* Rv3802 phospholipase. J. Biol. Chem. 293, 1363–1372. 10.1074/jbc.RA117.000240 29247008 PMC5787812

[B17] Gonzalez-y-Merchand J. A. Colston M. J. Cox R. A. (1999). Effects of growth conditions on expression of mycobacterial *murA* and *tyrS* genes and contributions of their transcripts to precursor rRNA synthesis. J. Bacteriol. 181, 4617–4627. 10419962 10.1128/jb.181.15.4617-4627.1999 PMC103595

[B18] Gupta R. S. Lo B. Son J. (2018). Phylogenomics and comparative genomic studies robustly support division of the genus *Mycobacterium* into an emended genus *Mycobacterium* and four novel genera. Front. Microbiol. 9:67. 10.3389/fmicb.2018.00067 29497402 PMC5819568

[B19] Jesus R. S. Piana M. Freitas R. B. Brum T. F. Alves C. F. S. Belke B. V.. (2018). *In vitro* antimicrobial and antimycobacterial activity and HPLC-DAD screening of phenolics from *Chenopodium ambrosioides* L. Braz. J. Microbiol. 49, 296–302. 10.1016/j.bjm.2017.02.012 29037505 PMC5914197

[B20] Kaur G. Sharma A. Narang T. Dogra S. Kaur J. (2018). Characterization of ML0314c of *Mycobacterium leprae* and deciphering its role in the immune response in leprosy patients. Gene 643:26–34. 10.1016/j.gene.2017.12.001 29208413

[B21] Kumar S. Mehra R. Sharma S. Bokolia N. P. Raina D. Nargotra A.. (2018). Screening of antitubercular compound library identifies novel ATP synthase inhibitors of *Mycobacterium tuberculosis*. Tuberculosis 108, 56–63. 10.1016/j.tube.2017.10.008 29523328

[B22] Liu D. Hao K. Wang W. Peng C. Dai Y. Jin R.. (2017). Rv2629 Overexpression delays *Mycobacterium smegmatis* and *Mycobacteria tuberculosis* entry into log-phase and increases pathogenicity of *Mycobacterium smegmatis* in mice. Front. Microbiol. 8:2231. 10.3389/fmicb.2017.02231 29187838 PMC5694894

[B23] Lopez M. Quitian L. V. Calderon M. N. Soto C. Y. (2018). The P-type ATPase CtpG preferentially transports Cd (2+) across the *Mycobacterium tuberculosis* plasma membrane. Arch. Microbiol. 200, 483–492. 10.1007/s00203-017-1465-z 29197950

[B24] Marney M. W. Metzger R. P. Hecht D. Valafar F. (2018). Modeling the structural origins of drug resistance to isoniazid via key mutations in *Mycobacterium tuberculosis* catalase-peroxidase, KatG. Tuberculosis 108, 155–162. 10.1016/j.tube.2017.11.007 29523317 PMC7330162

[B25] Marrakchi H. Bardou F. Lanéelle M.-A. Daffé M. (2008). A comprehensive overview of mycolic acid structure and biosynthesis, in The Mycobacterial Cell Envelope, eds Daffé M. Reyrat J-M. (Washington, DC: ASM Press), 41–62.

[B26] Milo R. Phillips R. (2016). Concentrations and absolute numbers, in Cell Biology by the Numbers, eds Milo R. Phillips R. (New York, NY: Garland Science), 147–151.

[B27] Mortuza R. Aung H. L. Taiaroa G. Opel-Reading H. K. Kleffmann T. Cook G. M.. (2018). Overexpression of a newly identified d-amino acid transaminase in *Mycobacterium smegmatis* complements glutamate racemase deletion. Mol. Microbiol. 107, 198–213. 10.1111/mmi.13877 29134701

[B28] Oren A. Garrity G. (2018). List of new names and new combinations previously effectively, but not validly, published. Int. J. Syst. Evol. Microbiol. 68, 1411–1417. 10.1099/ijsem.0.002711 31825780

[B29] Papagiannakis A. Niebel B. Wit E. C. Heinemann M. (2017). Autonomous metabolic oscillations robustly gate the early and late cell cycle. Mol. Cell 65, 285–295. 10.1016/j.molcel.2016.11.018 27989441

[B30] Richards S. J. Isufi K. Wilkins L. E. Lipecki J. Fullam E. Gibson M. I. (2018). Multivalent antimicrobial polymer nanoparticles target mycobacteria and Gram-negative bacteria by distinct mechanisms. Biomacromolecules 19, 256–264. 10.1021/acs.biomac.7b01561 29195272 PMC5761047

[B31] Roller B. R. Stoddard S. F. Schmidt T. M. (2016). Exploiting rRNA operon copy number to investigate bacterial reproductive strategies. Nat. Microbiol. 1:16160. 10.1038/nmicrobiol.2016.160 27617693 PMC5061577

[B32] Schindelin J. Arganda-Carreras I. Frise E. Kaynig V. Longair M. Pietzsch T.. (2012). Fiji: an open-source platform for biological-image analysis. Nat. Methods 9, 676–682. 10.1038/nmeth.2019 22743772 PMC3855844

[B33] Shasmal M. Sengupta J. (2012). Structural diversity in bacterial ribosomes: mycobacterial 70S ribosome structure reveals novel features. PLoS ONE 7:e31742. 10.1371/journal.pone.0031742 22384065 PMC3286452

[B34] Singh A. Vijayan M. Varshney U. (2018). Distinct properties of a hypoxia specific paralog of single stranded DNA binding (SSB) protein in mycobacteria. Tuberculosis 108, 16–25. 10.1016/j.tube.2017.10.002 29523318

[B35] Srivastava A. Asahara H. Zhang M. Zhang W. Liu H. Cui S.. (2016). Reconstitution of protein translation of *Mycobacterium* reveals functional conservation and divergence with the Gram-negative bacterium *Escherichia coli*. PLoS ONE 11:e0162020. 10.1371/journal.pone.0162020 27564552 PMC5001721

[B36] Tsaloglou M. N. Nemiroski A. Camci-Unal G. Christodouleas D. C. Murray L. P. Connelly J. T.. (2018). Handheld isothermal amplification and electrochemical detection of DNA in resource-limited settings. Anal. Biochem. 543, 116–121. 10.1016/j.ab.2017.11.025 29224732

[B37] Verma A. K. Sarin R. Arora V. K. Kumar G. Arora J. Singh P.. (2018). Amplification of Hsp 65 gene and usage of restriction endonuclease for identification of non-tuberculous rapid grower mycobacterium. Indian. J. Tuberc. 65, 57–62. 10.1016/j.ijtb.2017.08.030 29332650

[B38] Vijay S. Mukkayyan N. Ajitkumar P. (2014). Highly deviated asymmetric division in very low proportion of mycobacterial mid-log phase cells. Open Microbiol. J. 8, 40–50. 10.2174/1874285801408010040 24949109 PMC4062944

[B39] Vilvhéze C. Kremer L. (2017). Acid-fast positive and acid-fast negative *Mycobacterium tuberculosis*: The Koch paradox. Microbiol. Spectr. 5:TBTB2-0003-2015 10.1128/microbiolspec PMC11687472 28337966

[B40] Yamada H. Mitarai S. Chikamatsu K. Mizuno K. Yamaguchi M. (2010). Novel freeze-substitution electron microscopy provides new aspects of virulent *Mycobacterium tuberculosis* with visualization of the outer membrane and satisfying biosafety requirements. J. Microbiol. Methods 80, 14–18. 10.1016/j.mimet.2009.09.022 19799941

[B41] Yamada H. Yamaguchi M. Chikamatsu K. Aono A. Mitarai S. (2015). Structome analysis of virulent *Mycobacterium tuberculosis*, which survives with only 700 ribosomes per 0.1 fl of cytoplasm. PLoS ONE 10:e0117109. 10.1371/journal.pone.0117109 25629354 PMC4309607

[B42] Yamada H. Yamaguchi M. Shimizu K. Murayama S. Y. Mitarai S. Sasakawa C.. (2017). Structome analysis of *Escherichia coli* cells by serial ultrathin sectioning reveals the precise cell profiles and the ribosome density. Microscopy 66, 283–294. 10.1093/jmicro/dfx019 28854579

[B43] Yamaguchi M. (2006). Structome of *Exophiala* yeast cells determined by freeze-substitution and serial ultrathin sectioning electron microscopy. Curr. Trends Microbiol. 2, 1–12.

[B44] Yamaguchi M. Aoyama T. Yamada N. Chibana H. (2016a). Quantitative measurement of hydrophilicity/hydrophobicity of the plasma-polymerized naphthalene film (Super Support Film) and other support films and grids in electron microscopy. Microscopy 65, 444–450. 10.1093/jmicro/dfw031 27512014

[B45] Yamaguchi M. Chibana H. (2018). A method for obtaining serial ultrathin sections of microorganisms in transmission electron microscopy. J. Vis. Exp. 131:e56235. 10.3791/56235 29364224 PMC5908661

[B46] Yamaguchi M. Namiki Y. Okada H. Mori Y. Furukawa H. Wang J.. (2011). Structome of *Saccharomyces cerevisiae* determined by freeze-substitution and serial ultrathin-sectioning electron microscopy. J. Electron Microsc. 60, 321–335. 10.1093/jmicro/dfr052 21908548

[B47] Yamaguchi M. Okada H. Namiki Y. (2009). Smart specimen preparation for freeze substitution and serial ultrathin sectioning of yeast cells. J. Electron. Microscopy 58, 261–266. 10.1093/jmicro/dfp013 19289851

[B48] Yamaguchi M. Yamada H. Higuchi H. Yamamoto K. Arai Y. Murata S.. (2016b). High-voltage electron microscopy tomography and structome analysis of unique spiral bacteria from the deep sea. Microscopy 65, 363–369. 10.1093/jmicro/dfw016 27230559

[B49] Yamaguchi M. Yamada H. Uematsu K. Horinouchi Y. Chibana H. (2018). Electron microscopy and structome analysis of unique amorphous bacteria from the deep sea in Japan. Cytologia. [Epub ahead of print].

[B50] Yang K. Chang J. Y. Cui Z. Li X. Meng R. Duan L.. (2017). Structural insights into species-specific features of the ribosome from the human pathogen *Mycobacterium tuberculosis*. Nucleic Acids Res. 45, 10884–10894. 10.1093/nar/gkx785 28977617 PMC5737476

[B51] Zhu M. Dai X. (2018). On the intrinsic constraint of bacterial growth rate: *M. tuberculosis's* view of the protein translation capacity. Crit. Rev. Microbiol. 44, 455–464. 10.1080/1040841X.2018.1425672 29334314

