# Supplementary material for: Mycolicibacterium smegmatis, Basonym Mycobacterium smegmatis, Expresses Morphological Phenotypes Much More Similar to Escherichia coli Than Mycobacterium tuberculosis in Quantitative Structome Analysis and CryoTEM Examination
Source: Front Microbiol. 2018 Sep 11;9:1992. doi: 10.3389/fmicb.2018.01992 (PMC6145149; doi:10.3389/fmicb.2018.01992)
Supplement: Supplementary file 13 [file Table_1.PDF]

Table S1. Comparison of one-dimensional data obtained from serial ultrathin sectioning and cryoTEM of MSG and MTB.

			Number of cell examined	Diameter (μm)		Length (μm)	Aspect ratio
				OM	PM		
M. smegmatis	Serial ultrathin section	Average	7	0.58[a, c, h]	0.55[a, d]	3.54	6.27[f]
		SD		0.05	0.05	1.83	3.71
		Min		0.51	0.49	2.07	3.35
		Max		0.66	0.63	7.41	14.35
	CryoTEM	Average	61	0.77[b, h]	ND	3.46[e]	4.44
		SD		0.08	ND	1.40	1.69
		Min		0.66	ND	1.08	1.34
		Max		1.00	ND	6.27	7.65
M. tuberculosis [1]	Serial ultrathin section	Average	5	0.34[a, j]	0.30[a]	2.70	8.24[g]
		SD		0.03	0.02	1.07	3.61
		Min		0.29	0.27	1.30	3.61
		Max		0.37	0.33	3.80	13.00
	CryoTEM	Average	14	0.60[b, j]	ND	2.65[e]	4.38
		SD		0.06	ND	0.74	1.16
		Min		0.51	ND	1.43	2.60
		Max		0.73	ND	3.85	6.68
E. coli [2]	Serial ultrathin section	Average	9	0.89[c]	0.85[d]	2.47	2.84[f, g]
		SD		0.06	0.06	0.37	0.46
		Min		0.67	0.77	2.05	2.18
		Max		0.80	0.92	3.04	3.46

ND: not determined.
a: $p < 0.0001$, b: $p < 0.0001$, c: $p < 0.0001$, d: $p < 0.0001$, e: $p < 0.05$, f: $p < 0.02$, g: $p < 0.001$, h: $p < 0.000001$, j: $p < 0.000001$.

1: Yamada et al., 2015
2: Yamada et al., 2017
