# Supplementary material for: Mycolicibacterium smegmatis, Basonym Mycobacterium smegmatis, Expresses Morphological Phenotypes Much More Similar to Escherichia coli Than Mycobacterium tuberculosis in Quantitative Structome Analysis and CryoTEM Examination
Source: Front Microbiol. 2018 Sep 11;9:1992. doi: 10.3389/fmicb.2018.01992 (PMC6145149; doi:10.3389/fmicb.2018.01992)
Supplement: Supplementary file 14 [file Table_2.PDF]

Table S2. Comparison of OM and PM surface area between *M. smegmatis*, *M. tuberculosis* and *E. coli* in serial ultrathin sections

		OM (μm^2)	PM (μm^2)
M. smegmatis	Average	5.62[a]	5.10[b]
	SD	2.19	1.95
	Min	3.00	2.77
	Max	9.28	8.48
M. tuberculosis [1]	Average	3.03[a]	2.67[b]
	SD	1.33	1.19
	Min	1.20	1.10
	Max	4.33	3.78
E. coli [2]	Average	4.31	3.75
	SD	0.75	0.69
	Min	3.35	2.90
	Max	5.61	4.76

a: $p<0.05$
b: $p<0.05$

1: Yamada et al., 2015
2: Yamada et al., 2017
