# Supplementary material for: Mycolicibacterium smegmatis, Basonym Mycobacterium smegmatis, Expresses Morphological Phenotypes Much More Similar to Escherichia coli Than Mycobacterium tuberculosis in Quantitative Structome Analysis and CryoTEM Examination
Source: Front Microbiol. 2018 Sep 11;9:1992. doi: 10.3389/fmicb.2018.01992 (PMC6145149; doi:10.3389/fmicb.2018.01992)
Supplement: Supplementary file 15 [file Table_3.PDF]

Table S3. Comparison of volume data between *M. smegmatis*, *M. tuberculosis* and *E. coli*.

		Volume (fl)				
		Whole cell	OM	Periplasm	PM	Cytoplasm
M. smegmatis	Average	0.91[a]	0.01	0.10	0.03	0.77[b]
	SD	0.37	0.004	0.07	0.01	0.31
	Min	0.44	0.01	0.03	0.01	0.38
	Max	1.55	0.02	0.19	0.04	1.30
M. tuberculosis [1]	Average	0.29[a, c]	0.01	0.06	0.02	0.21[b, d]
	SD	0.11	0.003	0.02	0.01	0.09
	Min	0.18	0.002	0.03	0.01	0.11
	Max	0.43	0.01	0.07	0.02	0.31
E. coli [2]	Average	1.01[c]	0.01	0.15	0.02	0.90[d]
	SD	0.36	0.002	0.05	0.003	0.16
	Min	0.75	0.01	0.10	0.01	0.73
	Max	1.91	0.02	0.23	0.02	1.18

a: $p<0.005$
b: $p<0.005$
c: $p<0.00001$
d: $p<0.0005$

1: Yamada et al., 2015
2: Yamada et al., 2017
