# Supplementary material for: Mycolicibacterium smegmatis, Basonym Mycobacterium smegmatis, Expresses Morphological Phenotypes Much More Similar to Escherichia coli Than Mycobacterium tuberculosis in Quantitative Structome Analysis and CryoTEM Examination
Source: Front Microbiol. 2018 Sep 11;9:1992. doi: 10.3389/fmicb.2018.01992 (PMC6145149; doi:10.3389/fmicb.2018.01992)
Supplement: Supplementary file 16 [file Image_1.PDF]

Supplementary Figures

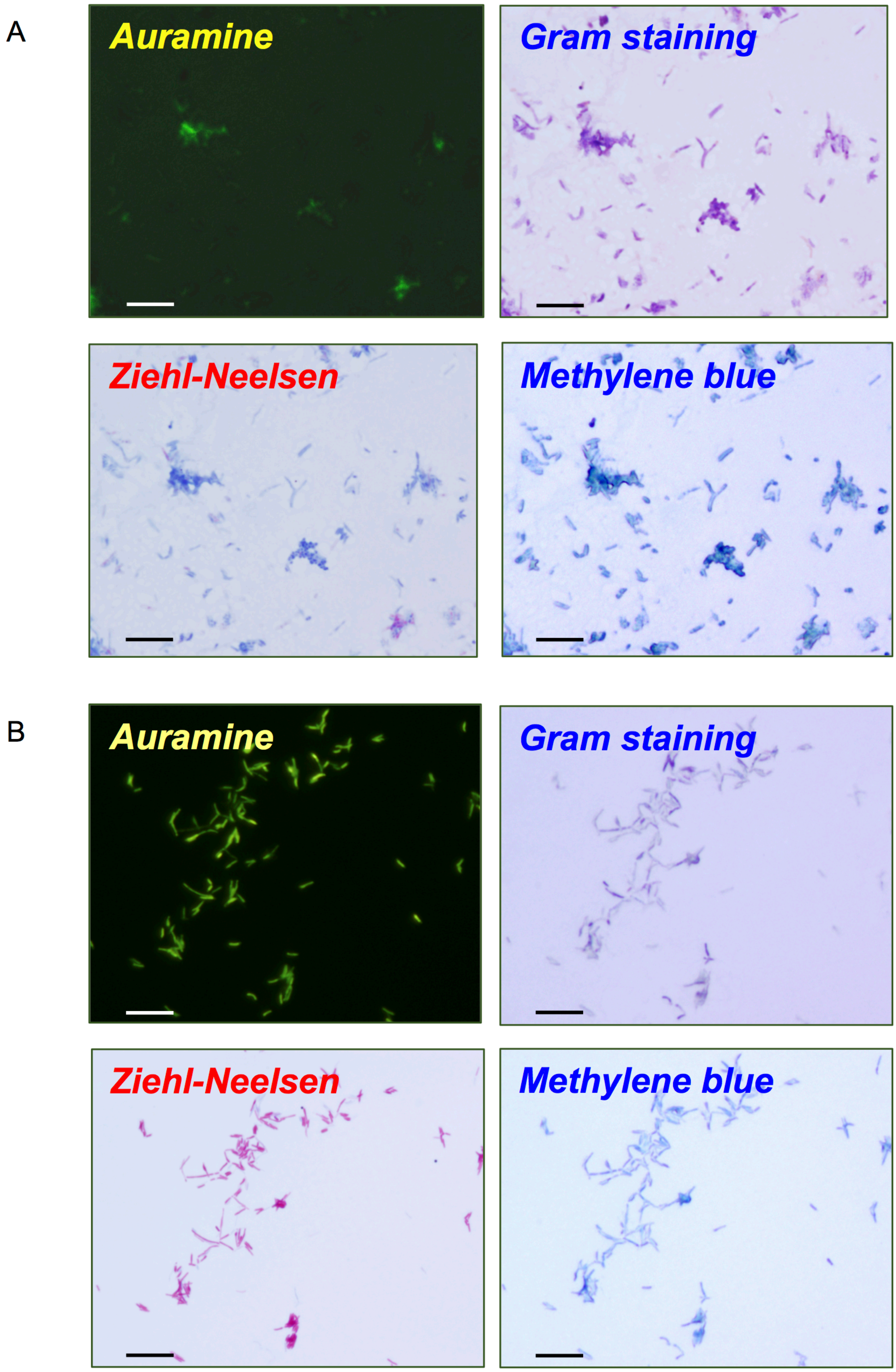

Figure S1. Auramine, Ziehl-Neelsen, methylene blue and Gram staining. Liquid-cultured *M. smegmatis* (A) and *M. tuberculosis* (B) cells on a smear were subjected to repeated staining and decolorization with auramine, Gram-staining, methylene blue and Ziehl-Neelsen staining in this order. All four images in each species were captured in the same field of view. Bar = 10 µm.
