# Supplementary material for: Mycolicibacterium smegmatis, Basonym Mycobacterium smegmatis, Expresses Morphological Phenotypes Much More Similar to Escherichia coli Than Mycobacterium tuberculosis in Quantitative Structome Analysis and CryoTEM Examination
Source: Front Microbiol. 2018 Sep 11;9:1992. doi: 10.3389/fmicb.2018.01992 (PMC6145149; doi:10.3389/fmicb.2018.01992)
Supplement: Supplementary file 17 [file Image_2.PDF]

Supplementary Figures

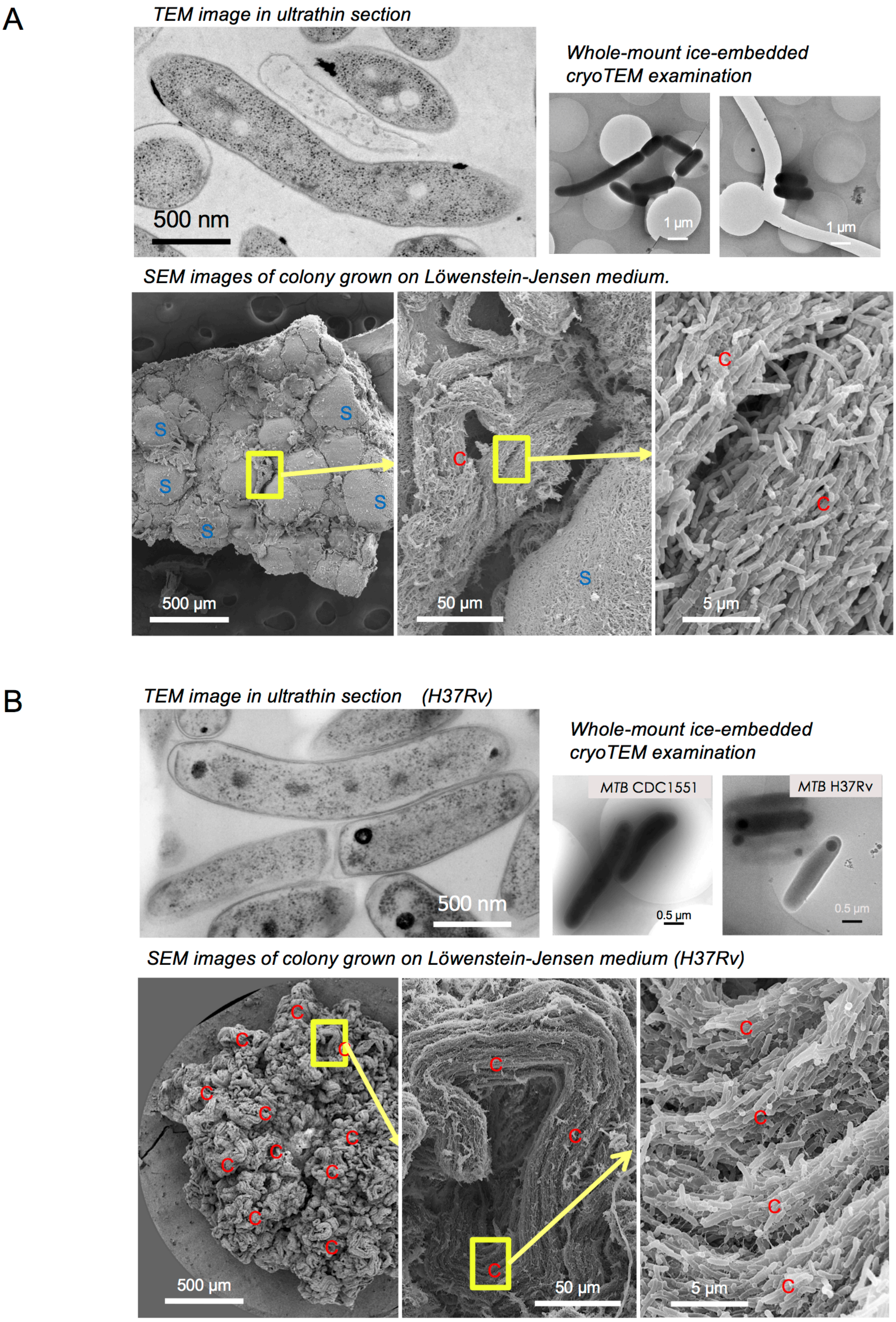

Figure S2. TEM, cryoTEM and SEM images of MSG and MTB. One part of liquid-cultured *M. smegmatis* (A) and *M. tuberculosis* (B, H37Rv and CDC1551) cells (Yamada et al., 2010 and Yamada et al., 2012) were processed through rapid-freeze and freeze-substation, and embedded in epoxy resin. Ultrathin sections were cut (left top panel in A and B). Another part of liquid-cultured MSG and MTB were fixed with glutaraldehyde and washed with phosphate buffer. The bacillary cells were quick-frozen and whole-mount ice-embedded samples were observed with cryoTEM (top right panel in A and B). Colonies grown on Löwenstein-Jensen solid medium were fixed with glutaraldehyde, post-fixed with 1% OsO_4, dehydrated with ethanol series, freeze-dried with *t*-butyl alcohol, and subjected to metal coating. Then, the samples were observed with SEM (JEOL, JSM-5800, AccV: 10kV) at three magnification. (bottom panel in A and B). S: smooth surface, C: cord formation.
