# Supplementary material for: Mycolicibacterium smegmatis, Basonym Mycobacterium smegmatis, Expresses Morphological Phenotypes Much More Similar to Escherichia coli Than Mycobacterium tuberculosis in Quantitative Structome Analysis and CryoTEM Examination
Source: Front Microbiol. 2018 Sep 11;9:1992. doi: 10.3389/fmicb.2018.01992 (PMC6145149; doi:10.3389/fmicb.2018.01992)
Supplement: Supplementary file 18 [file Data_Sheet_1.pdf]

Supplementary Material

Mycolicibacterium smegmatis, basonym Mycobacterium smegmatis, expresses morphological phenotypes much more similar to *Escherichia coli* than *Mycobacterium tuberculosis* in quantitative structome analysis and CryoTEM examination

Hiroyuki Yamada[1*], Masashi Yamaguchi[2], Yuriko Igarashi[1], Kinuyo Chikamatsu[1], Akio Aono[1], Yoshiro Murase[1], Yuta Morishige[1], Akiko Takaki[1], Hiroji Chibana[2], Satoshi Mitarai[1,3]

[1] Department of Mycobacterium Reference and Research, the Research Institute of Tuberculosis, Japan Anti-Tuberculosis Association, Kiyose, Tokyo, 204-8533, Japan
[2] Medical Mycology Research Center, Chiba University, Chiba, 260-8673, Japan.
[3] Department of Basic Mycobacteriology, Graduate School of Biomedical Sciences, Nagasaki University, Nagasaki, Japan.

*** Correspondence:**
Dr. Hiroyuki Yamada
hyamada@jata.or.jp

Running title: Structome analysis of *M. smegmatis*.

Keywords: *Mycolicibacterium smegmatis, Mycobacterium tuberculosis*, structome analysis, cryofixation, freeze-substitution, serial ultra-thin sectioning, transmission electron microscopy, ribosome enumeration.

Supplementary Videos

Supplementary Video S1: A total of 38 serial ultrathin sections for Cell 1 were shown in slide show.

Supplementary Video S2: A total of 44 serial ultrathin sections for Cell 2 were shown in slide show.

Supplementary Video S3: A total of 21 serial ultrathin sections for Cell 3 were shown in slide show.

Supplementary Video S4: A total of 19 serial ultrathin sections for Cell 4 were shown in slide show.

Supplementary Video S5: A total of 35 and 28 serial ultrathin sections for Cell 5 (right) and Cell 6 (left) were shown in slide show, respectively.

Supplementary Video S6: A total of 35 serial ultrathin sections for Cell 7 were shown in slide show.

Supplementary Video S7: 3-dimensional reconstruction of Cell 1 based on 38 serial ultrathin sections.

Supplementary Video S8: 3-dimensional reconstruction of Cell 2 based on 44 serial ultrathin sections.

Supplementary Video S9: 3-dimensional reconstruction of Cell 3 based on 21 serial ultrathin sections.

Supplementary Video S10: 3-dimensional reconstruction of Cell 4 based on 19 serial ultrathin sections.

Supplementary Video S11: 3-dimensional reconstruction of Cell 5 (red) and Cell 6 (purple) based on 35 and 28 serial ultrathin sections, respectively.

Supplementary Video S12: 3-dimensional reconstruction of Cell 7 based on 35 serial ultrathin sections.

References

Yamada, H., Mitarai, S., Chikamatsu, K., Mizuno, K., Yamaguchi, M. (2010). Novel freeze-substitution electron microscopy provides new aspects of virulent *Mycobacterium tuberculosis* with visualization of the outer membrane and satisfying biosafety requirements. *J Microbiol Methods*. 80, 14-18. doi: 10.1016/j.mimet.2009.09.022.

Yamada, H., Bhatt, A., Danev, R., Fujiwara, N., Maeda, S., Mitarai, S., Chikamatsu, K., Aono, A., Nitta, K., Jacobs, W. R., Jr., and Nagayama, K. (2012). Non-acid-fastness in *Mycobacterium tuberculosis ΔkasB* mutant correlates with the cell envelope electron density. *Tuberculosis*. 92, 351-357. doi: 10.1016/j.tube.2012.02.006.
